# Validation of the Parkinson's Disease Caregiver Burden Questionnaire in Progressive Supranuclear Palsy

**DOI:** 10.1155/2021/9990679

**Published:** 2021-05-10

**Authors:** Martin Klietz, Hannah V. Eichel, Selma Staege, Anna Kutschenko, Gesine Respondek, Meret K. Huber, Stephan Greten, Günter U. Höglinger, Florian Wegner

**Affiliations:** ^1^Department of Neurology, Hannover Medical School, Carl-Neuberg-Straße 1, Hannover 30625, Germany; ^2^Center for Systems Neuroscience, Hannover 30559, Germany

## Abstract

Progressive supranuclear palsy (PSP) is an atypical Parkinson syndrome with axial akinetic-rigid symptoms, early postural instability, and ocular motor impairments. Patients experience a rapid loss of autonomy and care dependency; thus, caregivers must assist in the activities of daily living early in the course of the disease. Caregiver burden is an extremely important factor in disease management. However, there are no specific questionnaires for assessment of caregiver burden in PSP. This study aims to validate the Parkinson's disease caregiver burden questionnaire (PDCB) as a specific measure of caregiver burden in PSP. PSP patients were assessed by the PSP rating scale, PSP quality-of-life questionnaire (PSP-QoL), Montreal cognitive assessment test (MoCA), and geriatric depression scale (GDS-15). Caregivers filled out the short form 36-health survey, GDS-15, PDCB, and the caregiver burden inventory (CBI). 22 patient caregiver pairs completed the study. PDCB showed a highly significant correlation with the CBI (r 0.911; *p* < 0.001). Internal reliability of the PDCB measured by Cronbach's alpha was favourable at 0.803. These data support the specificity of the PDCB in PSP caregivers. Future studies with larger sample sizes of PSP patients and caregivers and a multicentric longitudinal design should be performed to gain further insight of caregiver burden in PSP.

## 1. Introduction

Progressive supranuclear palsy (PSP) is a rare neurodegenerative disease which can be histopathologically classified as 4-repeat tauopathy [1]. Patients affected by this movement disorder present with axial rigidity, akinesia, early postural instability, frontal-dysexecutive syndrome, and a large variety of further symptoms [2, 3]. In contrast to Parkinson's disease (PD), patients with 4-repeat tauopathy hardly ever respond to dopamine replacement therapy or other symptomatic treatments [3]. Patients with PSP suffer from loss of autonomy and care dependency early in the course of this disease [4]. These symptoms correspond to severe restrictions in patients' health-related quality of life [5]. Furthermore, PSP caregivers experience a more profound burden as compared to PD caregivers due to greater symptom load earlier in disease progression [4].

Caregiver burden can be measured by non-disease-specific questionnaires such as the caregiver burden inventory (CBI) [4]. Recently, a PD-specific questionnaire (PDCB) for caregiver burden was established and validated [6, 7]. As caregiver burden negatively affects patient outcomes in PD and PSP, validation of a disease-specific caregiver burden questionnaire in crucial. Studies using a disease-specific questionnaire might allow more insight into disease-related burden of caregivers in PD and also atypical Parkinsonism.

The aim of this monocentric cross-sectional observational study is to investigate the validity of the German version of the PDCB for the assessment of caregiver burden in PSP diagnosed by the new MDS criteria in comparison with an established non-disease-specific questionnaire.

## 2. Methods

### 2.1. Participants

Ethical approval was obtained from the local Ethics Committee of Hannover Medical School (No. 3178–2016, Amendment in 2019). In this monocentric cross-sectional observational study, PSP patients with the clinical diagnosis “suggestive of “, “possible” or “probable” PSP according to the Movement Disorder Society criteria in 2017 [8] and their caregivers were screened for participation in the Department of Neurology, Hannover Medical School. 42 PSP patients were screened for participation in the study from May 2019 until August 2020. Of these screened PSP patients, 5 did not have a caregiver, 8 were in institutional care, and 7 refused to participate in the study. Our final sample included 22 PSP patients and their corresponding caregivers; all participants gave their written informed consent. Inclusion criteria were (1) a PSP diagnosis confirmed by a movement disorder specialist in accordance with the Movement Disorder Society criteria 2017 and (2) PSP patients with a primary caregiver. Exclusion criteria were (1) idiopathic Parkinson's disease or other atypical Parkinsonism and (2) PSP patients with professional caregivers (e.g., nurses in an institutional care facility) as these professional caregivers were responsible for major parts of PSP patient caregiving. In all PSP patients, a movement disorder specialist confirmed the diagnosis. The participating PSP patients and their caregivers received no compensation for participation in this study. Overall, the completion of the examination and questionnaires took about one hour for the patient and 30 minutes for the caregiver.

### 2.2. Measures

Caregivers and patients were asked to provide general background and demographic information (e.g., patients' disease duration as well as the daily amount of caregiving time).

Patients were examined by using the PSP rating scale for the assessment of PSP symptoms [9] and the Montreal Cognitive Assessment (MoCA) as a cognitive screening test ranging from 0 to 30 points [10]. A score of 30 to 26 points in the MoCA was considered as normal cognitive function, of 25 to 21 points as mild cognitive impairment, and below 21 points as indicative of dementia [10]. Patients' health-related quality of life (HR-QoL) was evaluated by the PSP-QoL questionnaire consisting of 45 items measuring the physical (22 items) and mental (23 items) impact of PSP [11]. Note that the total score for the PSP-QoL questionnaire ranges from 0 to 180 points and was converted to 0 to 100 percent. Patients with higher PSP-QoL scores display lower HR-QoL. Caregivers were asked to assist patients with cognitive impairment in completing the PSP-QoL questionnaire to ensure correct results and avoid anosognosia (as described in [12]).

HR-QoL of the caregivers was measured by the short form 36-health survey (SF-36). This questionnaire measures HR-QoL across eight dimensions [13]. Subscale scores were percentage-transformed so that a score of 0 would indicate maximum impairment, and a score of 100 would suggest the absence of any reported impairment. Since all subscale scores were highly correlated with each other, we calculated an average score across the eight scales as also carried out in other studies of our group [12].

Caregiver and patient depressive mood symptoms were assessed by the geriatric depression scale in its 15-item version (GDS-15), and values of 6 or more were defined as manifest depression [14, 15].

Caregivers of the PSP patients were asked to complete the German version of the Parkinson's disease caregiver burden questionnaire (PDCB) and the caregiver burden inventory (CBI) [6, 16]. Both questionnaires and the German translation process were extensively described in a recent publication. In brief, two independent native speakers translated the PDCB and CBI forward and backward, and after that, an internal group of movement disorder specialists discussed the wording and confirmed the correctness of the translation. The PDCB contains 20 items that can be answered on a 5-point Likert scale ranging from 0 to 4. Participants can, thus, reach a maximum of 80 points on this questionnaire. In addition, respondents are asked to rate their global burden as a caregiver on a scale from 0 to 100. The total PDCB score is obtained by dividing this global burden rating by 5 and adding it to the PDCB questionnaire sum score. The total PDCB result can range from 0 to 100 with higher scores indicating higher caregiver burden.

The CBI consists of 22 items on a 0-to-4 5-point Likert scale. Caregivers can score a maximum of 88 points on the CBI suggesting high caregiver burden.

### 2.3. Analyses

Demographic characteristics of the patients and informal caregivers are displayed as mean ± standard deviation (SD) and minimal and maximal value. The internal reliability of the PDCB in caregivers of PSP patients was defined by Cronbach's alpha. To examine the validity of the PDCB total score compared with the CBI sum score, PSP-QoL, PSP rating scale, GDS-15 of the patient and caregiver, MoCA of the patient, caregiving hours per day, and SF-36 average score of the caregivers, the Spearman correlation was calculated. This form of analysis yielded comparable results to the former validation studies [7]. To correct for multiple testing, the level of significance was set to *α* = 0.05/*n* (with *n* being the number of analyzed predictors for the correction of multiple comparisons). For interpretation of Spearman correlations, a correlation coefficient of 0.01–0.19 was considered as no or negligible relationship, 0.2–0.29 weak relationship, 0.3–0.39 moderate relationship, 0.40–0.69 strong relationship, and >0.69 very strong relationship [17]. Statistical calculations were carried out using SPSS 25.0 (IBM, Armonk, NY).

## 3. Results

### 3.1. Patient and Caregiver Characteristics

All characteristics of the 22 PSP patients and their caregivers are displayed in Table 1. On average, PSP patients were 69.1 years old (SD ± 6.3 min 53, max 78) with a disease duration of 5 years (SD ± 2.3 min 2, max 11). The cohort of recruited PSP patients consisted of 86% (*n* = 19) Richardson's syndrome and 14% (*n* = 3) PSP with other phenotypes according to Movement Disorder Society diagnostic criteria 2017 [8]. The PSP non-Richardson's syndrome group (*n* = 3) contained PSP patients with predominant Parkinsonism (PSP-P, *n* = 1), predominant corticobasal syndrome (PSP-CBS, *n* = 1), and with progressive gait freezing (PSP-PGF + PSP-P, *n* = 1) [18].

With regard to disease severity, patients scored on average 38.3 points in the PSP rating scale total score (SD ± 16.5 min 15, max 69). The overall estimation of cognitive function, as indicated by the mean MoCA score of 21.6 (SD ± 5.1 min 9, max 29), suggested mild impairment in PSP patients. In total, cognition was impaired in 18 patients, of which 7 reached the MoCA criterion suspicious for dementia (<21 points). The GDS-15 score with a mean of 5.7 (SD ± 4.1 min 0, max 15) indicated a tendency for mild symptoms of depression in PSP patients. In our sample, 9 PSP patients met criteria for depression according to the GDS-15 cutoff. Hence, the GDS-15 cutoff for depression is not validated for PSP patients [14, 15]. Regarding HR-QoL, PSP patients presented a mean PSP-QoL total score of 23.3 (SD ± 16.6 min 6.1, max 79.9), suggesting limited HR-QoL in general.

Caregivers were younger than the PSP patients with a mean age of 62.3 years (SD ± 10.8 min 40, max 81). Most of the caregivers were spouses (81.8%) and only a minority were a child or child-in-law of the patient. The caregivers spent on average 7.4 hours per day (SD ± 6.5 min 1, max 24) on caregiving for their patient. In PSP caregivers, moderate burden was found based on the CBI (mean 32.4, SD ± 14.5 min 10, max 61) and PDCB (mean 34.6, SD ± 15.6 min 6, max 61). All subscores (e.g., physical burden, sleep disturbance, patient symptoms, responsibilities, patient medication, social burden, and patient and self-relationship) of the PDCB are displayed in Table 2.

Depressive symptoms, as assessed by GDS-15, were mild in our cohort of PSP caregivers with a mean score of 3.3 (SD ± 3.6 min 0, max 13). However, 4 of the participating caregivers were diagnosed as clinically depressive (GDS-15 score ≥6).

Internal reliability and convergent validity of Parkinson's disease caregiver burden questionnaire for caregivers of PSP patients.

PDCB as specific and CBI as general measures of caregiver burden showed a highly significant correlation even after correction for multiple comparison (Table 3). Further known outcome parameters, such as PSP rating scale, MoCA performance of the patient, and GDS-15 scores of the patient and caregiver showed a significant correlation with caregiver burden measured by the PDCB as a specific caregiver burden questionnaire for PD. However, these correlations were not significant after correction for multiple comparisons. Interestingly, the caregivers' HR-QoL estimated by total SF-36 scores was not significantly correlated with caregiver burden measured by the PDCB.

By comparing regression coefficients between the PDCB and the CBI, we found that the PDCB scores showed slightly higher correlation coefficients with outcome measures of the PSP patients (e.g., PSP rating scale, MoCA, PSP-QoL, and GDS-15). In contrast, the CBI scores were associated with higher correlation coefficients of caregiver parameters, such as depressive mood, HR-QoL, and caregiving hours per day.

The internal consistency of the PDCB measured by Cronbach's alpha was found to be 0.803. Only item two “assistance in activities of daily living” of the PDCB showed a high level of variance and decreased the internal reliability of the scale (Table 4). Cronbach's alpha increased to 0.825 when item two was deleted. None of the other items led to a relevant change in Cronbach's alpha.

## 4. Discussion

In this study, we analyzed the validity of the PDCB in caregivers of PSP patients. Overall, the PDCB correlated with most caregiver-related outcome measures. These results support the application of the PDCB in PSP patients to detect caregiver burden.

Patients suffering from PSP experience fastly progressing symptoms including parkinsonian, dystonic, and cognitive features which are insufficiently treatment responsive [19]. These motor and nonmotor symptoms lead to rapid decrease in HR-QoL, progressive loss of autonomy, and high mortality [20, 21]. Compared to PD, the caregiver of a PSP patient has to support the activities of daily living early in the course of the disease. However, due to symptom severity, homebound care cannot be maintained very often. Usually, frequent falls lead to hospitalization and care dependency of the patients with Richardson's syndrome [22]. Caregiver burden in PSP is a very important issue because its prevention or reduction might sustain caregivers' quality of life, and thus, institutionalization of the patient may be avoided.

Until now, no specific scale for caregiver burden in PSP is available. The caregiver burden inventory (CBI) is an established generic measurement for caregiver burden. This questionnaire is not disease specific, allowing data to be compared beyond boundaries of certain diagnoses. However, in diseases with an extremely varied course and specific complications, like in PSP, a more specific questionnaire for the detection of caregiver burden might prevent loss of information and assist to elucidate all aspects of caregiving. Pillas et al. developed a specific instrument (PQoL Carers) to measure HR-QoL of the caregivers of patients with atypical Parkinsonism [23]. However, despite correlation with caregiver burden measured by the CBI, the PQoL Carers only assesses caregivers` HR-QoL and not caregiver burden in particular.

The PDCB is positively validated for early and advanced PD [24]. This questionnaire showed a significant correlation with PD-specific outcome measures, such as the MDS-UPDRS, the PDQ-39, and PDQ-8 version in recent validation studies and PD cohorts [25–27]. According to our data, the PDCB offers a new opportunity to measure specific burden in caregivers of patients suffering from PSP. In our PSP cohort, the PDCB also showed a good correlation with most outcome measures of caregiver burden. The PSP rating scale as a measure of disease severity displayed a moderate correlation with both the PDCB and CBI. Interestingly, the PDCB showed higher correlation coefficients with patient factors of caregiver burden than the CBI. Since the generic CBI showed slightly higher correlation coefficients with caregiver outcome parameters, the combination with the PDCB would help to avoid loss of information in future studies. These data fit to the recent finding that the PDCB also correlated significantly with the MDS-UPDRS parts I–IV in a large PD cohort [25].

Both, PDCB and CBI correlated with the MoCA score of our PSP patients. Several studies also described a correlation of cognitive function and caregiver burden in PD patients [28, 29]. Generally, in PSP as well as in our cohort, a high proportion of patients suffer from mild cognitive impairment or dementia. This cognitive decline of patients seems to be a specific driver of caregiver burden in PSP [30]. Interestingly, Schmotz et al. did not find a significant correlation of cognitive function measured by the Mini Mental Status Examination (MMSE) and caregiver burden in a cohort of PSP patients [4]. By looking closer into cognitive subdomains of the MoCA, the PDCB was correlated specifically with attentional and language impairments of PD patients in a German cohort [25]. Since this PDCB scale was predominantly designed for PD, the stronger correlation of caregiver burden with motor symptoms of PD and PSP patients seems adequate.

In many studies of various groups, depressive symptoms of PD patients correlated with caregiver burden, mostly measured by generic questionnaires ([31] for review). In a recent study of our group, we could confirm this finding with the PDCB in a cohort of PD patients. Our current data in PSP patients and caregivers also showed a significant correlation of depressive symptoms measured by the GDS-15 and caregiver burden indicated by the PDCB. On the contrary, a recent study of Schmotz et al. did not detect a significant correlation of depressive symptoms of PSP patients and caregiver burden which might be due to the limited sample size or application of the longer-scale version GDS-30 [4]. In line with our data, Zhong et al. [24] also reported a significant correlation of both patient and caregiver depressive symptoms measured by the hospital anxiety and depression scale, with caregiver burden estimated by the PDCB [24]. Taken together, depressive mood of the PSP patient and caregiver is likely to have an impact on caregiver burden. However, the number of participants and the specific measure of depressive symptoms in a particular study might interfere with the extent of this finding.

The PDCB also correlated significantly with most caregiver outcome parameters, including caregiver depression. Surprisingly, HR-QoL of the caregiver did not correlate with the PDCB which might be due to the limited sample size and the generic measurement of HR-QoL by the SF-36. We speculate that a significant correlation would have been detected if a more specific PQoL Carers questionnaire had been used [23]. However, when designing the study, we decided to use the generic SF-36 because of better comparability to recent studies of our group [12, 32]. In other studies, it was suggested that caregivers with massive burden also experienced reduced HR-QoL [33, 34].

The amount of caregiving time per day is an important indicator for caregiver burden ([31] for review). In our study, we found only a tendency for this correlation that did not reach significance, mainly because of the high variance of the individual hours of caregiving. To our knowledge there were no data available on time spent on caregiving and burden of care in PSP. However, it seems rational to assume that this association might also be found in PSP caregivers in studies with a larger sample size, since it could be described in other movement disorders such as dystonia and PD [12]. In future studies, the newly adapted caregiver task questionnaire will help to gain more insights in this topic [26].

In the planning phase of this study, we identified 2 items of the PDCB that might be unspecific for patients with PSP. These items emphasize impulse control disorders (item 13) as well as tremor and dyskinesia (item 17) [7]. Nevertheless, these items were rated very low by the caregivers and, therefore, did not influence the internal reliability measured as Cronbach's alpha. Interestingly, the support of activities of daily living (item (2) showed a high level of variance, reducing the internal reliability of the PDCB. This might be caused due to the heterogeneous impairments of the participating PSP patients.

### 4.1. Limitations

An important limitation of this study is that the PDCB is not designed and constructed for the detection of caregiver burden on caregivers of PSP patients. However, we chose the PDCB because of the excellent comparability of the data with Parkinson's disease caregivers. Furthermore, the new definition of PSP with several new phenotypes prevented us from designing a new caregiver burden questionnaire because it seems nearly impossible to cover specific caregiver impairments of all different phenotypes and their varied longitudinal trajectory in one questionnaire. We report cross-sectional data from a monocentric validation study. However, longitudinal data would be also desirable. In this validation study, we were able to investigate only 22 PSP patients and their caregivers due to the rarity of the disease, early institutionalization of the patients, and the monocentric setting. The only moderate number of participants may explain the missing correlation of the caregiver's health-related quality of life and caregiver burden. In future studies, we plan to investigate caregiver burden in a longitudinal and multicentric setting.

## 5. Conclusions

Our study supports the validity of the PDCB in caregivers of patients with PSP and promotes the general applicability of the PDCB in Parkinsonism. Future modifications of the PDCB might help to increase the specificity of the PDCB for PSP caregivers. Further studies with larger patient cohorts are needed to analyze the trajectories of caregiver burden during the disease course in the different PSP subtypes.

## Figures and Tables

**Table 1:** Patient (*n* = 22, 13 females) and caregiver (*n* = 22, 12 females) characteristics.

	Mean ± SD	Min	Max
PSP patients			
Age (years)	69.1 ± 6.3	53	78
Disease duration (years)	5 ± 2.3	2	11
PSP rating scale	38.3 ± 16.5	15	69
PSP-QoL	23.3 ± 16.6	6.1	79.9
MoCA	21.6 ± 5.1	9	29
GDS-15	5.7 ± 4.1	0	15

Caregivers			
Age (years)	62.3 ± 10.8	40	81
Relationship to PSP patients	81.8% spouse living together with the patient and 18.2% child/child-in-law		
Caregiving hours per day	7.4 ± 6.5	1	24
CBI	32.4 ± 14.5	10	61
PDCB (total)	34.6 ± 15.6	6	61
SF-36 (total)	66.5 ± 19.2	32.3	91.7
GDS-15	3.3 ± 3.6	0	13

**Table 2:** Subscores of the Parkinson's disease caregiver burden questionnaire (*n* = 22).

	Mean ± SD	Min	Max
Physical burden (2 items)	2.5 ± 1.8	0	6
Sleep disturbance (2 items)	2.5 ± 2.3	0	7
Patient symptoms (5 items)	7.8 ± 3.3	3	13
Responsibilities (3 items)	4.2 ± 2.3	0	9
Patient medications (2 items)	1.2 ± 1.5	0	4
Social burden (3 items)	2.5 ± 2.3	0	8
Patient- and self-relationship (3 items)	5.3 ± 2.1	2	9
Global burden (1 item 0–100)	45.6 ± 24.1	0	80
PDCB total (0–100)	34.6 ± 15.6	6	61

**Table 3:** Spearman correlations of patient and caregiver measures including Parkinson's disease caregiver burden questionnaire total score (*n* = 22).

	1	2	3	4	5	6	7	8	9
1. PDCB total score	—								
2. CBI	0.911^*∗∗*^	—							
3. PSP-QoL	0.408	0.359	—						
4. PSP rating scale	0.585^*∗*^	0.552	0.450	—					
5. Patient GDS-15	0.471^*∗*^	0.373	0.533	0.292	—				
6. Patient MoCA	−0.462^*∗*^	−0.453	−0.198	−0.654	−0.247	—			
7. Caregiving hours per day	0.05	−0.048	−0.184	0.168	−0.473	0.11	—		
8. Caregiver SF-36	−0.474	−0.584	−0.193	−0.371	−0.228	0.252	−0.221	—	
9. Caregiver GDS-15	0.535^*∗*^	0.542	0.396	0.345	0.236	−0.119	0.087	−0.802	—

Note: correlations and *r* values of interest for the validity of the PDCB total score are displayed in column 1. The remaining correlations of the other column were not compared to a significance threshold. ^*∗*^Significance at *p* < 0.05; ^*∗∗*^significance at *p* < 0.006.

**Table 4:** Item characteristics of the German version of the Parkinson's disease caregiver burden questionnaire (*n* = 22).

Item	Mean	SD	Corrected item total correlation	*α* if item 2 was deleted
1	1.00	1.34	0.602	0.779
2	1.48	1.54	−0.053	0.825
3	1.00	1.27	0.499	0.786
4	1.48	1.29	0.527	0.784
5	2.05	1.40	0.423	0.791
6	2.14	1.11	0.150	0.806
7	1.05	1.24	0.511	0.785
8	0.76	1.22	0.188	0.805
9	1.81	1.17	0.549	0.784
10	0.76	0.94	0.207	0.802
11	1.57	1.21	0.383	0.793
12	1.90	1.30	0.543	0.783
13	0.19	0.402	0.510	0.796
14	1.05	1.32	0.273	0.800
15	0.76	0.70	0.593	0.788
16	1.05	1.12	0.794	0.770
17	0.67	1.16	0.141	0.807
18	0.67	1.20	0.320	0.797
19	3.19	1.03	0.513	0.787
20	1.48	1.17	0.143	0.807

## Data Availability

Data are available on reasonable request to the corresponding author. The English and German versions of the PDCB are included in the original open access publications. To avoid redundancy, the authors did not include these versions in the actual manuscript.

## Abbreviations

CBI: Caregiver burden inventory
GDS-15: Geriatric Depression Scale (15-item)
MoCA: Montreal cognitive assessment
PDCB: Parkinson's disease caregiver burden inventory
PSP: Progressive supranuclear palsy
PSP rating scale: Progressive supranuclear palsy rating scale
PSP-QoL: PSP quality-of-life questionnaire
SD: Standard deviation
SF-36: Short form 36-health survey of the caregiver.
